# Multi-Rule Based Ensemble Feature Selection Model for Sarcasm Type Detection in Twitter

**DOI:** 10.1155/2020/2860479

**Published:** 2020-01-09

**Authors:** Karthik Sundararajan, Anandhakumar Palanisamy

**Affiliations:** Department of Information Technology, Madras Institute of Technology, Anna University, Chennai–603202, Tamilnadu, India

## Abstract

Sentimental analysis aims at inferring how people express their opinion over any piece of text or topic of interest. This article deals with detection of an implicit form of the sentiment, referred to as sarcasm. Sarcasm conveys the opposite of what people try to convey in order to criticize or ridicule in a humorous way. It plays a vital role in social networks since most of the tweets or posts contain sarcastic nuances. Existing approaches towards the study of sarcasm deals only with the detection of sarcasm. In this paper, in addition to detecting sarcasm from text, an approach has been proposed to identify the type of sarcasm. The main motivation behind determining the types of sarcasm is to identify the level of hurt or the true intent behind the sarcastic text. The proposed work aims to improve upon the existing approaches by incorporating a new perspective which classifies the sarcasm based on the level of harshness employed. The major application of the proposed work would be relating the emotional state of a person to the type of sarcasm exhibited by him/her which could provide major insights about the emotional behavior of a person. An ensemble-based feature selection method has been proposed for identifying the optimal set of features needed to detect sarcasm from tweets. This optimal set of features was employed to detect whether the tweet is sarcastic or not. After detecting sarcastic sentences, a multi-rule based approach has been proposed to determine the type of sarcasm. As an initial attempt, sarcasm has been classified into four types, namely, polite sarcasm, rude sarcasm, raging sarcasm, and deadpan sarcasm. The performance and efficiency of the proposed approach has been experimentally analyzed, and change in mood of a person for each sarcastic type has been modelled. The overall accuracy of the proposed ensemble feature selection algorithm for sarcasm detection is around 92.7%, and the proposed multi-rule approach for sarcastic type identification achieves an accuracy of 95.98%, 96.20%, 99.79%, and 86.61% for polite, rude, raging, and deadpan types of sarcasm, respectively.

## 1. Introduction

Natural language processing (NLP) deals with incorporating the computer with the capacity of understanding the language of human beings just like how it is spoken. NLP falls under the domain of artificial intelligence (AI). The major challenge in developing NLP-based systems is that the human language is not always precise due to its complex linguistic structure and the difficulty in interpretation. Slangs, dialects, and the context play a huge role in making it a little difficult for computers to understand. Recent developments in NLP can be attributed to the fact that vast amount of information has been made available due to the huge impact of social media in the recent years. Sentiment can be considered as a viewpoint that an individual might possess. It is an idea or feeling which is held by a person. It is a combination of feelings and opinions which forms the basis for an action. Human sentiments play a major role in their day-to-day activities. Sentiment analysis is a use case of NLP. Sentiment analysis is generally used by data scientists to assess comments available in social media to determine how a particular business or brand is performing. For example, the reviews of a product can be analyzed to modify the shortcomings and identify the areas of improvements required in the product which in turn can lead to a business growth.

Sarcasm can be considered as an implicit form of sentiment. It usually conveys the opposite of what has been intended. Sarcasm is generally associated with irony and satire or wit that is used to refute, insult, make fun of, or amuse. For example, the teacher exclaimed “Kudos to your hard work. I have never been more impressed in my life. Lol!” A plain look at this sentence may reveal that it is an appreciation. However, the context and the body language of a speaker indicate the sarcastic nature of this expression. In the absence of visible expressions, determining sarcasm in a tweet is a challenge. An interesting perspective of sarcasm was provided by Deliens et al. [1] where the analysis was conducted upon two sarcastic conditions: egocentric and allocentric. The former term indicates that the sarcasm was felt or observed only in the participant's perspective and not from addressee's point of view and the latter indicating sarcasm being observed from both participant's and addressee's point of view. The generic interpretation of results conveys that the prosodic features, the ones involving patterns of stress and sounds, are more helpful in detecting sarcasm than contextual features.

Consider the example: “*Oh how I love being ignored #sarcasm*.” While this tweet has a hashtag to indicate sarcasm, it is not necessary for a person to always include hashtags to indicate sarcasm. This poses an additional challenge to extract the right features and characteristics of tweets based on which sarcastic tweets can be identified. It is important in areas like sentiment analysis and affective computing since sarcasm can totally flip the polarity of a sentiment even though it may look different. Basic analysis of sentiments from texts might not be efficient to understand the clear motivation due to the presence of various literary devices such as sarcasm, irony, etc. [2]. Hence detecting sarcasm is very essential in order to avoid any sort of misunderstanding in any type of communication and to ensure that meanings intended in the statements are understood as it is. Manually detecting sarcasm can be a tedious process which can be simplified by automated sarcasm detection and analysis. Detecting sarcastic statements has become a vital task in social media applications as it influences the organizations that mine social media information. Despite the presence of various possible features that can be extracted from text, they can be grouped into broad categories, namely, lexical, pragmatic, hyperbolic, and contextual features [3].

The key focus of this research is to categorize sarcasm into various types which helps to understand the level of hurt or intent to hurt that is present in the sarcastic statement. As sarcasm can invoke a wide range of feelings in a person, it can either create a feeling of fun for the receiver or at the worst case, it can even invoke a deep sense of emotional hurt. The application of type detection can be helpful in understanding the emotions behind sarcasm which in turn can even give an insight into the emotional state of the people involved in a sarcastic conversation, i.e., the person who uses sarcasm and the person upon whom sarcasm was intended. Deeper analysis can even give insight into the relationship between the emotional state of a person and the type of sarcasm that he or she employs during that time. Such levels of understanding will improve the process of sarcasm detection. Initially, an ensemble-based feature selection approach has been proposed to identify the optimal set of features needed to detect sarcasm.

The main contribution of this work is the classification of sarcasm into 4 types, in addition to detecting sarcasm. In order to detect and classify, a multi-rule based approach has been proposed. Along with these, an attempt has been made to model the mood change based on the type of sarcasm exhibited by the user. Experimental results showed that the proposed method obtained encouraging results in detecting and categorizing sarcasm. The major objectives of this research work include the following:

1. To classify sarcasm into various types based on the emotional aspects, thereby determining whether the emotional state of a person influences the type of sarcasm exhibited by them
2. To determine the optimal set of features essential for sarcastic type classification with proposed ensemble learning algorithm
3. To propose a multi-rule based approach for classifying sarcasm into various types in order to tackle the problem of vagueness and uncertainty exhibited by natural languages

## 2. Related Work

### 2.1. Literature Survey on Feature Extraction

Ravi and Ravi [4] came up with an ensemble text feature selection method in order to identify sarcasm and irony from reviews and news articles. An AUC value of 91.46% for satiric news and AUC of 88.86% for ironic reviews were recorded. Bouazizi and Ohtsuki [5] developed a system for detecting sarcasm and thereby intended to demonstrate that detection of sarcasm increases the performance of sentiment analysis. Khokhlova et al. [6] suggested the Twitie software for tokenizing and tagging microblog text and the Sketch Engine System for classifying words into eight emotions: Anger, Anticipation, Disgust, Fear, Joy, Sadness, Surprise, and Trust. Rockwell [7] used the speech data utterances and performed acoustic analysis and perceptual coding and found that the former performed better than the latter in discriminating sarcasm and nonsarcasm. Ragini et al. [8] developed an approach for generating disaster response with sentiment analysis. The authors used POS and lexicon features and observed that the accuracy was 30% more than historical methods. El-Masri et al. [9] applied a sentiment analysis tool in Arabic tweets for polarity detection. The authors used machine learning and lexicon methods in order to analyze the polarity of the tweets. Neppalli et al. [10] developed a system for identifying the polarity of tweets during Hurricane Sandy. The system achieved an accuracy of 75.91%, and the major issue was the lack of connection between the disaster and people who tweeted about it. Kim [11] put up a system which uses the relevance theory method to provide more analytic account by adding cognitive explanations. Yoo et al. [12] proposed a system for detecting real-time events from social media. The authors performed sentiment analysis using convolutional networks. The accuracy was about 84%, and as the number of tweets increased, the model observed a gradual decrease in the accuracy.

Musoff [13] used categorical falsity as metaphor and the contradictory as irony/sarcasm to find out the distinction between irony and sarcasm. The author was able to deduce that there was a clear difference between irony, sarcasm, and metaphor. Ren et al. [14] developed a method by dividing the process into two approaches: one by integrating key contextual information (CANN-KEY) and the other by integrating all contextual information (CANN-ALL). It avoided manual feature engineering and used a real-valued word vectors which resulted in F-measures of 56.37 and 62.05, respectively. Lei et al. [15] developed a system to predict emotions by generating emoticon and part of speech (POS) tagging on news articles. The accuracy was found to be 63.57%. Ghaissi and Lee [16] developed a feature selection module which was domain dependent. Vectorized tweets were taken as input which resulted in highly sparse input matrix. It was found that the sentiment analysis increased sensitivity but domain-specific nature of the process made it difficult to generalize beyond the chosen target. Xiong et al. [17] performed a multi-level sentiment analysis and as a result, enriched the method of word embedding as word level information was integrated with tweet level information. Karoui et al. [18] employed supervised learning approach and used 4 features for identifying irony. Reliability of hashtags and pragmatic features had to be ensured, and the accuracy was about 72.36%. Cai et al. [19] developed a framework based on ensemble text feature selection method for detecting sarcasm, irony, and satire from reviews and news articles. The logistic regression method was found to achieve higher accuracy values of 91.46% and 88.86% in detection of satiric and ironic reviews, respectively. Jiménez-Zafra et al. [20] employed a supervised learning and lexicon based sentiment analysis. Kim et al. [21] developed an ensemble regularization method by combining three regression models. Standard misclassification rate was found to be 0.001.

### 2.2. Literature Survey on Sarcasm Detection

Bharti et al. [22] processed real-time tweets by using Flume and Hive. The authors detected sarcasm by developing a hidden Markov model-based algorithm and MapReduce algorithm. It can be implemented with or without Hadoop Framework where it was found out that the time taken without Hadoop was 11609 s while time taken with Hadoop was 4147 s. Mukherjee and Bala [23] suggested a system which used Naïve Bayes for classification and fuzzy c-means for clustering. Since context words had been used, it was found out to be more effective than methods which used content words alone. The accuracy was found out to be 65%. A small dataset of only 2000 tweets was used; hence, Naïve Bayes worked well. Voyer and Vu [24] developed a model to experiment how negative literal statements would affect sarcasm detection in auditory perception. The approach achieved 80% accuracy in sarcasm identification. Bhan and D'silva [25] suggested a system to measure sarcasm using different algorithms such as Naive Bayes, logistic regression, and linear regression where scores generated for each algorithm were compared to present the most efficient way. The linear SVC model obtained precision, recall, and F-score values of 0.86, 0.87, and 0.86, respectively. Chaudhari and Chandankhede [26] considered a rule-based method for hashtag tokenization, sarcasm detection, and polarity detection which also included statistical, distributional, and deep learning classification techniques. The SVM classifier of statistical approach achieved a precision of 64% and a recall of 39%, while the distributional semantic models achieved a 7%–10% F-score where it was found that there were not enough feature sets to explore datasets. Dharwal et al. [27] made use of various sarcasm analyzing techniques for filtering sarcastic statements from the text.

Gupta et al. [28] came up with a mood swing analyzer using k-means clustering to perform sentiment analysis. The approach attained an accuracy of 85.03%. Lagerwerf [29] developed a system to detect irony and sarcasm from advertisements and public announcements. AL-Sharuee et al. [30] developed a system for sentiment analysis using contextual analysis and unsupervised ensemble learning in order to handle domain-dependency problem. The average accuracy for many datasets was found to be 82.45%. Persicke et al. [31] developed a system for teaching autistic children a method for detecting sarcasm and responding to the same. The author included rules along with video clips for this purpose. Dynel [32] made use of various competing academic approaches to detect sarcasm and irony. Ager [33] developed a method to detect sarcasm based on assumptions about the speaker and the addressed topic. Deliens et al. [1] developed a noncontextual strategy for sarcasm detection. It was found out to be an efficient strategy but was time consuming. Gent [34] used a chip to design the system to cope with huge volume of data which can monitor thousands of twitter accounts for tweets that mention a specific phrase. Energy efficiency was posing a problem. Fernández-Caballero et al. [35] suggested a system to detect the emotional state of patients by analyzing their behavior, physiological signals, and expressions. Porshnev et al. [36] developed a system to analyze the twitter user's mood and thereby predict the stock price movement. Zhang et al., [37] used network regulators in microblog text to analyze the sentiment. The network regulators analyzed public opinions and made decision regarding the sentiment. Xiaomei et al. [38] used sentiment consistency and emotional contagion for sarcasm detection. The developed model was able to outperform baseline methods consistently and significantly but time consumption was high.

Schuch et al. [39] used congruency sequence effect and a hanker interface paradigm and stroop-like interface for identifying conflict adaptation. Song et al. [40] used convolutional networks for sentiment classification framework. It achieved good results when compared to the state-of-the-art techniques. The method heavily relied on the performance of the pretrained saliency detection networks. Fardoun et al. [41] collected information from student moods using RFID tags. It turned out to be time consuming, and effectiveness was also under question. Swami et al. [42] developed an English-Hindi coded dataset for the purpose of sarcasm detection. A total of 5250 tweets were used, and *n*-grams and sarcastic hashtags were used as features. Random forest was used for classification and achieved an accuracy of 78.4%. Rajeswari and ShanthiBala [43] discussed about recognizing sarcastic emotion from individuals. Though information about sarcastic types was listed in the article, implementation and experimentation details were not provided regarding sarcasm detection as well as categorization.

This highlights the importance of the proposed work where the problem of categorizing sarcasm has been attempted, and the results have been provided with experimental evidence.

### 2.3. Literature Survey on Fuzzy and Rough Set

Tran et al. [44] developed a rough set system from perspective information, and it was observed to perform better than the traditional greedy methods. Tiwari et al. [45] suggested knowledge extraction in framing expert and intelligent systems which transformed fuzzy decision system (FDS) into intuitionistic FDS (IFDS) with a fixed degree of hesitancy. The presence of significant amount of noise was the major issue in IFDS. Hiai and Shimada [46] came forth with a system that classified sarcastic tweets using three-stage judgement process based on rules, boosting rules, and rejection rules. The approach classified tweets into 8 classes which were more promising than the baseline model as the precision rate and recall rate were found to be 0.028 and 0.543. Fang and Hu [47] developed a system based on fuzzy implicators and co-implicators for fuzzy granules. The uncertainty measures, reductions of the granular variable, and the application of theoretical results are yet to be explored deeply. Qian et al. [48] combined the neighborhood and local rough set approaches. Approximation and attribute reduction algorithm was performed with limited labeled dataset. The authors conducted a rough set-based data analysis with limited labeling and evaluated the performance of the approach using various datasets.

Overall, the major limitations of the existing approaches include the following: most of the existing works focus primarily on lexical and syntactic feature-based approaches, lack of efficient techniques to handle the uncertain and vague aspects of natural language data, presence of noises in the raw twitter data, etc.

## 3. Proposed Approach

**Figure 1:**
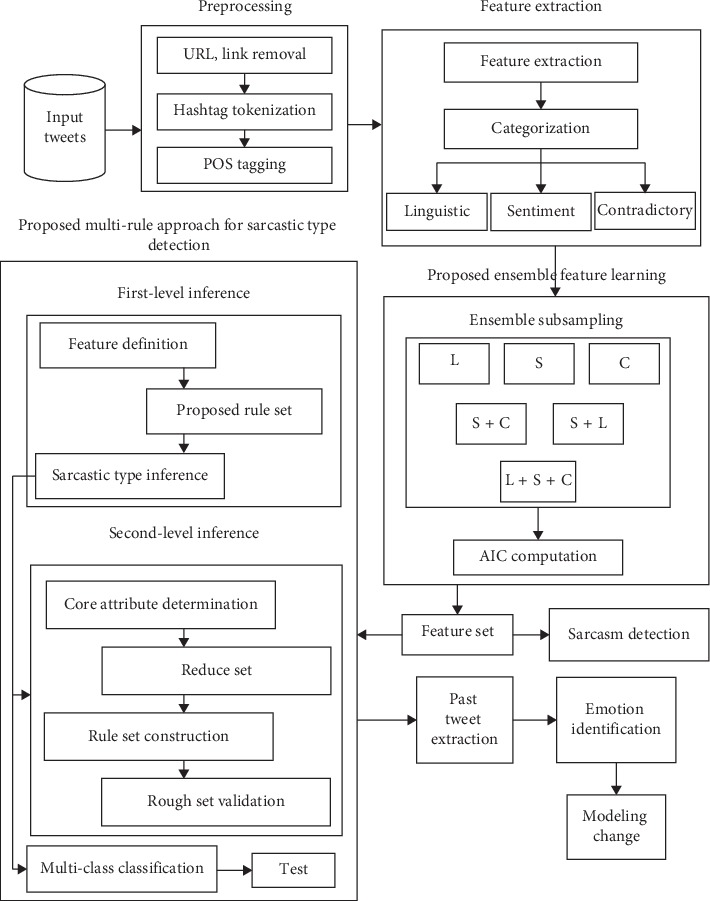
Proposed architecture diagram for sarcastic type detection.

The major objective of the proposed work is to identify if a given sentence is sarcastic or nonsarcastic with an optimal set of features, identified by the proposed ensemble feature selection. These features would be leveraged to further classify the sarcastic statement to a specific type with the proposed multi-rule based type classification approach. The results are validated using various classification algorithms. This research has attempted to find the correlation between the types of sarcasm and the possible mood changes.  Figure 1 describes the overall architecture of the proposed work.

Given a set of tweets “t,” the objective is to classify a particular tweet as sarcastic or nonsarcastic. The sarcastic tweets are then passed on to the proposed multi-rule based classification framework where it will be classified into its appropriate type.

(1) $$\begin{array}{c} \text{SD}\left(\text{t}\right)⟶\left\{\text{S , NS}\right\},\\ \forall \text{S},\text{MRC}\left(\text{S}\right)⟶\left\{\text{Polite Sarcasm, Rude Sarcasm, Raging Sarcasm, Deadpan Sarcasm}\right\}, \end{array}$$

where SD ⟶ sarcasm detection; S ⟶ sarcastic class; NS ⟶ nonsarcastic class; and MRC ⟶ multi-rule based classification framework.

### 3.1. Data Acquisition and Preprocessing

Twitter API was used for the purpose of data collection. Twitter is one of the most popular social media platforms. People commonly use twitter for sharing their opinion, views, anger, displeasure, and all sorts of opinions or emotions about any event that makes it so popular. Tweets from Twitter are predominantly utilized in various fields of natural language processing applications. Tweets are obtained through Twitter API (Tweepy and Twython). Tweets are extracted on the basis of the following hash tags: #sarcasm, #sarcastic, #Sarcasm, and #notSarcasm. A total of 76,799 tweets are used for experimentation purpose. The tweets that are non-English are filtered out. The data from the twitter as such might be incomplete, inconsistent, and likely to possess many errors. Hence, the raw data obtained need to be cleaned and then transformed into an understandable format for further processing. Hashtags, URLs, and links are removed in preprocessing Algorithm 1 followed by which POS tagging, stemming, and lemmatization are performed to obtain understandable data.

### 3.2. Feature Extraction

Feature extraction has a huge role in determining the outcome of any machine learning task. The quality of classification, both qualitatively and quantitatively, depends on the features selected. This section, at a high level, focuses on extracting the features from tweets that can be categorized into various types, namely, lexical, hyperbolic, pragmatic, sentiment, and contradiction. Lexical features include *n*-gram, bigram, and unigram which are combination of words that are extracted from the tweets to aid in tokenization. Intensifiers are also identified as they might help in the sarcasm detection process. Pragmatic features like emoticon and smileys are extracted. The proposed system extracts a total of 20 features: noun and verb count, positive intensifier, negative intensifier, bigram, trigram, skip gram, unigram, emoji sentiment, sentiment score, interjections, punctuators, exclamations, question mark, uppercase, repeat words count, positive word frequency, negative word frequency, polarity flip, and parts of speech tagging.

Various sentiment-based features are extracted from the tweets like positive words frequency—total number of positive words; negative words frequency—total number of negative words; positive intensifiers—intensifiers exhibiting positive emotion; negative intensifiers—intensifier exhibiting negative emotion; *n* grams—set of consecutively occurring “*n*” words(*n* = 1(unigram); *n* = 2(bigram), etc.); and skip gram—*n* grams with an additional factor called skip distance; passive aggressive count gives the indirect expression of hostile intention; sentiment score gives the sentiment value in which a “−1” indicates negative sentiment and a “+1” indicates a positive sentiment; emoticon sentiment gives the polarity of the emoticon; polarity flip gives the reverse polarity of sentiment; the co-occurring terms need not be consecutive as the tokens can be skipped based on the skip distance value; noun and verb counts can be obtained from POS tagging of a tweet; POS Tagging is a way to tag each word present in the tweet with its appropriate parts of speech; exclamations and question marks are most meaningful among the various punctuators for detecting sarcasm. Uppercase words are extracted as features because sometimes people use capital lettered words to stress on the things that they want to convey strongly. These are the prominent set of features which will be useful for sarcasm detection. Once these features are extracted, a numerical value for the features is obtained. These extracted features are categorized into different groups such as linguistic, sentiment based, and contradiction based feature sets.  Table 1 represents a sample of trigram feature extracted from the input file. Once the features are extracted, they are then passed on to the proposed ensemble feature selection module, where the optimal set of features for detecting sarcasm will be identified.  Algorithm 2 explains about the extraction of aforementioned features in a brief manner.

### 3.3. Proposed Ensemble Feature Selection

This section aims at identifying the optimal set of features from the extracted feature set that can help in identifying a sarcastic tweet. Ensemble learning aims at finding and selecting the best set of features that will aid in accurate sarcasm detection. Various classification algorithms are also leveraged in identifying the optimal set of features that is sufficient enough to identify a sarcastic tweet. Once the features are extracted, sarcasm is detected by training and testing for individual features. The results of classification for individual features are compared. The features are then grouped into various categories such as linguistic features, contradictory features, and sentiment-based features. Ensemble is done for each category of features. Further ensemble is carried out with combination of categories of features. Models are obtained by training using the various classifiers like Random Forest, Naive Bayes, Support Vector Machine, K-Nearest Neighbor, Gradient Boosting, AdaBoost, Logistic Regression, and Decision Tree.

The Classification algorithms listed here are chosen based on the detailed analysis of literatures involving classification tasks. These algorithms have exhibited better performances, and after various experiments, the results obtained by these algorithms were found to be better. Multiple classification algorithms help in validating the performance of the sarcasm classification. Initially, the dataset is annotated with a “1” for all the sarcastic tweets and a “0” for all the nonsarcastic tweets. The annotation has been carried out manually and with the help of hashtags too. The annotated dataset after preprocessing is passed on to feature extraction module. Once the features are extracted, it is passed over to the classification module where different classification algorithms were applied to detect sarcasm. The aforementioned features were fed as input to classification algorithms, and based on those features, a tweet has been classified into sarcastic and nonsarcastic. The performance of different algorithms for classifying sarcasm has been discussed in the result section. Once the tweets are classified into sarcastic and nonsarcastic, feature ensembling has been proposed to identify the optimal set of features for classifying sarcasm into various types. The best set of features that provide a better accuracy for the aforementioned classifiers is selected for determination of type of sarcasm (Algorithm 3).

The results obtained in the previous step for feature set model selection are validated by Akaike information criterion (AIC). AIC is a measure to compute the quality of a particular model with respect to every other model under consideration. AIC is based upon the theory that a model would be of higher quality if there is a minimal information loss. If the AIC value for a particular feature is less, then it is more prominent. The AIC value is computed by

(2) $$\begin{array}{c} \text{AIC}=2k-\ln\left(\text{L}\right), \end{array}$$

where *k* represents the number of estimated parameters in the model and likelihood function is denoted by L. Assume that there are “R” models and the Akaike information criterion values of each models are given by AIC_1_, AIC_2_, AIC_3_, ..., AIC_R_. Then, the expression

(3) $$\begin{array}{c} \exp\left(\frac{\left({\text{AIC}}_{\min}-{\text{AIC}}_{i}\right)}{2}\right), \end{array}$$

denotes the probability that the *i*^th^ model might minimize the information loss.

This concept is applied in the selection of the features and the classifier for detection of sarcasm (Algorithm 4).

### 3.4. Proposed Multi-Rule Based Sarcastic Type Detection

This section involves developing a fuzzy system based on the part of the features identified in the previous module in order to classify the type of sarcasm for every sarcastic tweet. Fuzzy module estimates how much a particular sarcastic tweet belongs to a particular type of sarcasm based on the various membership functions defined as part of fuzzy rule-based system (FRBS). Rough set theory deals with vague, imprecise, and inconsistent data. After developing a fuzzy system, the rough set model is developed to determine the sufficiency and effectiveness of classification of type of sarcasm.

#### 3.4.1. Fuzzy Rule System

Fuzzy logic deals with systems with ambiguity or vagueness. Real-world applications may not be suited for binary solutions always. There may be instances where approximate logic would be preferred to crisp logic. It is in such cases that fuzzy logic exhibits its usefulness. The specialty of fuzzy logic is that it handles uncertainties to a good extent. It employs a “degree of truth” to all factors rather than plain “true” or “false” cases. Fuzzy associates a membership value to every element in the set. Consider a statement which says “The probability of a bottle containing wine is 0.7” which usually employs that there is a 70% chance that there is a bottle that contains wine. Now consider the statement which goes like “The membership value of a bottle containing wine is 0.7” which means that the bottle will contain wine up to 70% of its entire volume. This is basically the difference between fuzzy logic and probability.

A fuzzy set, A, can be represented as

(4) $$\begin{array}{c} A=\left\{\left(x,{µ}_{A}\left(x\right)\right)\left|x\in \text{U}\right.\right\}, \end{array}$$

where “*x*” is any element, “*μ*” represents the membership function, and “U” denotes the universal set.

**Figure 2:**
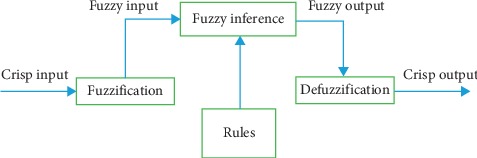
Fuzzy system architecture.

Fuzzy logic accomplishes task with fuzzy sets which represents a linguistic variable. Initially, a set of input and output variables are identified. Once the variables are identified, then linguistic variables are set for all the input variables. Linguistic variables contain linguistic hedges which signify various approximate states of the corresponding input and output. Once the variables are set, then the fuzzification process is performed with the help of membership functions which represents the fuzzy set in a graphical form. Then rules are formulated. Fuzzy logic is governed by IF-THEN rules. Fuzzy Inference System then evaluates the rules and finally performs the defuzzification task using one or more defuzzifying techniques. The aforementioned processes are described in Figure 2.

The various steps involved in defining a fuzzy logic system is described below (Algorithm 5):

  Fuzzificiation: the process of converting crisp inputs into fuzzy inputs
  Rule engine: this involves the development of IF-THEN rules that operate on the fuzzy variables
  Inference engine (also known as the controller): this step involves the execution of various fuzzy rules and determining the associated membership based on the input values
  Defuzzification: the process in which the resulting fuzzy output is converted into crisp outputs.

#### 3.4.2. Fuzzy Rule-Based Sarcastic Type Detection

In this work, a fuzzy rule-based system is developed with the features identified in the previous module to determine the type of sarcasm. In this work, we have attempted to classify sarcasm into four basic types: Polite, Rude, Raging, and Deadpan.

Polite is a form of sarcasm where the sarcastic sentence is more positive in nature. For example, “Wow, I love being ignored!!”

Rude is a form of sarcasm in which the sarcastic sentence contains moderately negative implications. For example, “I can explain it to you but I cannot understand it for you!”

Raging is an extension of rude sarcasm but is highly negative in the way of expression. For example, “Person1: You made me feel like the most horrible person in the world. Person 2: Yeah I made you feel like yourself.”

Deadpan is a type of sarcasm which is implicit in nature and is difficult to say if it is positively or negatively implied. For example, “I am confused in a good way!”

Based on the proposed ensemble learning method, the following set of features is extracted as the best set of features for sarcasm detection and are utilized for determining the type of Sarcasm. The features identified are as follows:

- Positive intensifier
- Negative intensifier
- Positive word count
- Negative word count
- Sentiment score
- Emoji sentiment

**Figure 3:**
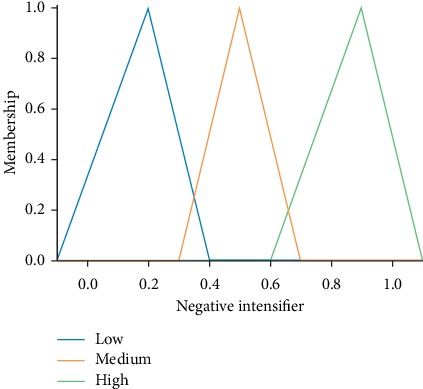
Fuzzy input variable.

These features are set as the antecedents in fuzzy rules. The fuzzy set for these terms is defined.  Figure 3 represents the fuzzy definition (membership function) for one of the input variables.

**Figure 4:**
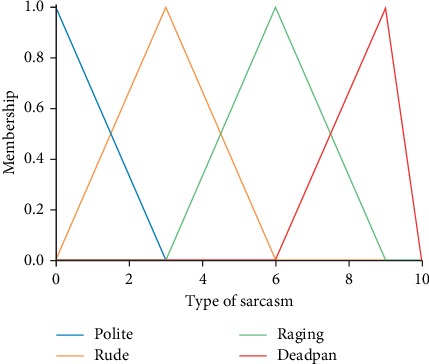
Fuzzy output variable.

Type of sarcasm is set as the consequent and can take one of the following values: Rude, Raging, Polite, and Deadpan.  Figure 4 describes the membership function for one of the output variables.

Membership functions are defined for the various fuzzy variables. After defining the membership functions with features as inputs and type of sarcasm as output, fuzzy rules are constructed. Examples of rules that are constructed for evaluation are shown below:

  rule7 = ctrl.Rule(PositiveIntensifier[‘High'] &NegativeIntensifier[‘Low'] & (PositiveWordCount[‘VeryHigh']|PositiveWordCount[‘High']|PositiveWordCount[‘Medium']| PositiveWordCount[‘Low']|PositiveWordCount[‘VeryLow']) &(SentimentScore[‘VeryHigh']) &NegativeWordCount[‘Low'] &EmojiSentiment[‘Medium'], TypeOfSarcasm[‘Polite'])
  rule24 = ctrl.Rule((NegativeIntensifier[‘Medium']|NegativeIntensifier[‘Low']) & (PositiveWordCount[‘Low']|PositiveWordCount[‘VeryLow']) & (NegativeWordCount[‘Low']|NegativeWordCount[‘VeryLow']|NegativeWordCount[‘Medium']) &SentimentScore[‘Low'] &EmojiSentiment[‘High'], TypeOfSarcasm[‘Rude'])

The rules described above are sample rules for detecting polite sarcasm and rude sarcasm. For every sarcastic tweet, the controller evaluates the input values with various linguistic hedges associated with membership function to decide the output. The output obtained is defuzzified to in order to get the crisp output, which is the type of sarcasm in this case.

**Figure 5:**
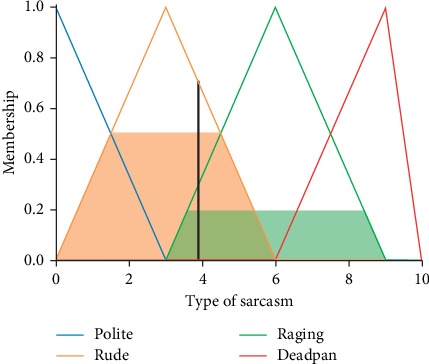
Sample output based on fuzzy rules.

Figure 5 shows an example of an output determined using the fuzzy logic system for a particular input combination.

#### 3.4.3. Rough Set-Based Sarcastic Type Detection

In addition to the fuzzy rule-based type detection, the proposed approach employs rough set rule set-based validation in order to strengthen and validate the classification process. Rough set approach is generally applied for the problems which has inadequate or approximate information and to perform decision making over it. It can be applied in order to obtain knowledge abstraction followed by which decision can be arrived upon. It involves developing an association rule generator that searches through the entire dataset and finds the rules that are sufficient to reveal the nature and frequency of relationship between the various data elements. Rough set-based knowledge discovery has certain important merits as in discovery of hidden patterns in the dataset, finding the relationships which cannot be found using statistical methods, finding out a set of data that is minimal and adequate enough for classification (data reduction), obtaining a set of decision rules from data, etc.

Assume a class of data, C; the definition of rough set for this class C is given by two approximations: lower approximation of C and upper approximation of C. Rough sets involve determining the minimal set of features or attributes which are sufficient enough and more prominent for representing knowledge. This reduced attribute set is referred to as a reduct set. The attribute set common to all reducts is referred to as the core. Rough set decision making involves identification of minimal set of rules that are sufficient enough for modelling the system.

**Figure 6:**
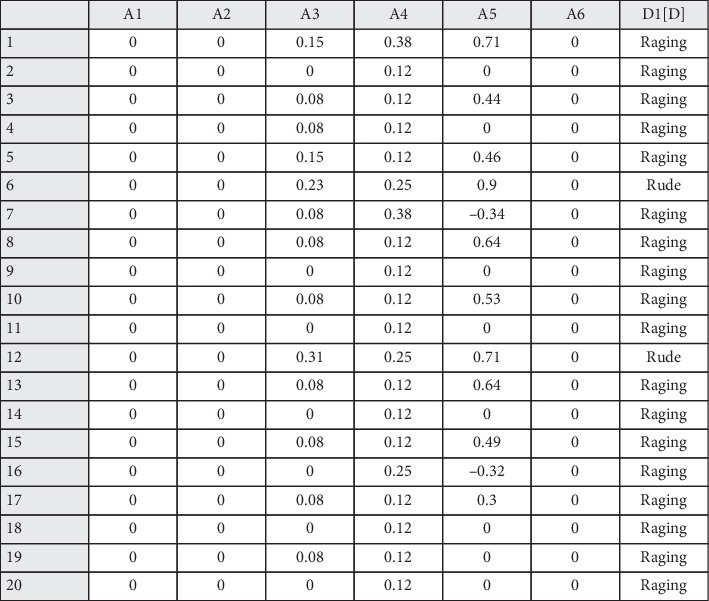
Sample snapshot of rough set attributes and decision making.

The fuzzy rule set-based classification has been validated based on the rough set approach. The type of sarcasm as predicted by the fuzzy rules and the feature set from the rules act as inputs for rough set analysis. The implementation is performed in ROSE2. As an initial step, the input data file is converted into.isf format in ROSE2. There were no missing values in the dataset.  Figure 6 shows the feature attributes and the decision attributes applied for analysis.

In Figure 6, columns A1 to A6 represent the features selected out of the proposed ensemble learning and last column denotes the result of the classification. Reducts are identified on the dataset and the rules are generated based on extended minimal covering. Classification of data is carried out using the generated decision rules. Clustering analysis is carried out on the type of sarcasm dataset obtained from the fuzzy logic rule set. This is used in validating the correctness of classification and the assumption made on the number of clusters in the data set (Algorithm 6)

#### 3.4.4. Modelling User's Mood Change

This section deals with the study of emotion or sentiment associated with the past tweets of user before and after a sarcastic tweet. The mood change is predicted to show how a sarcastic statement influences or reflects the mood of the person and vice versa. An attempt has been made in this work to model the change in mood based on the type of sarcasm. To predict mood change based on sarcastic type, a series of history of tweets for various users are extracted. The mood of the user is identified based on the past tweets of a user, and the mood changes for various types of sarcasm are studied. The changes in mood based on sarcastic type are modelled as a graph for individual people. The rest of this section addresses how the aforementioned steps for mood analysis of tweets are implemented (Algorithm 7).

The implementation of the mood analysis of tweets is carried out with NLTK and TextBlob. A sample set of sarcastic tweets for different types of sarcasm is selected. The past tweets of the user associated with these tweets are extracted. Feature extraction and classification of type of sarcasm, as described in the previous sections, are performed. For individual users, the history of tweets is extracted from either using the username or the user_id of the user. The polarity and sentiment score for these tweets are analyzed using TextBlob and NLTK. The change in mood is modelled as positive or negative change. The fluctuation in mood is predicted using the change and modelled as graph for individual person.

## 4. Results and Discussion

**Figure 7:**
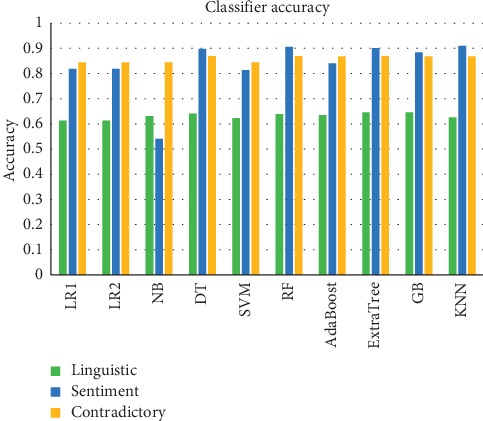
Classification accuracy of various models for feature set categories.

Around 76,799 tweets were used for experimentation purpose. The data are split into training and testing with 80 : 20 ratio. For the purpose of training, all the sarcastic tweets are annotated with a 1 and nonsarcastic tweets are annotated with a 0. Once preprocessing of tweets is done as mentioned in Section 3.1, feature extraction is carried out. Features that were extracted include bigram, trigram, skip gram, missing values, positive intensifier, negative intensifier, positive word count, negative word count, emoji sentiment, etc. The main goal of the proposed work is to get an optimal feature set for detecting sarcasm from the text input. The features were trained and tested to evaluate its performance. Further, the features were grouped into three categories, namely, textual, emotion based, and contrast based. Each category of feature is ensembled by using different classifiers such as Support Vector Machines, Gradient Boosting, Logistic Regression, AdaBoost, Random Forest, K-Nearest Neighbour, Naive Bayes, Decision Tree, and Bagging. It is found that sentiment-based features provide better predictability of sarcasm followed by the contradictory feature set as shown in Figure 7. It is observed that Random Forest yields better accuracy for various features.

**Figure 8:**
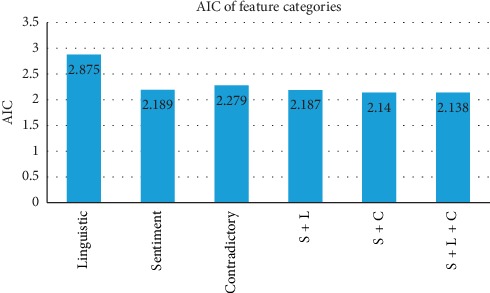
AIC value for feature set categories.

From Figure 7, it is clear that out of all feature categories, sentiment feature category (emotion) and the contradictory feature set achieve highest accuracy value. Hence, the optimal feature set among the extracted feature set comprises of sentiment features and contradictory features. This helps to ascertain the correctness and comprehensiveness of selected features identified by ensemble learning for sarcasm detection. The AIC value calculation, as shown in Figure 8, demonstrates that the combination of sentiment and contradictory features has the least AIC value, thereby leading to inference that these features can better model sarcasm detection. S denotes sentiment feature set; L denotes linguistic feature set; and C denotes contradictory feature set. The performance of the proposed system is compared with few of the baseline approaches.

**Figure 9:**
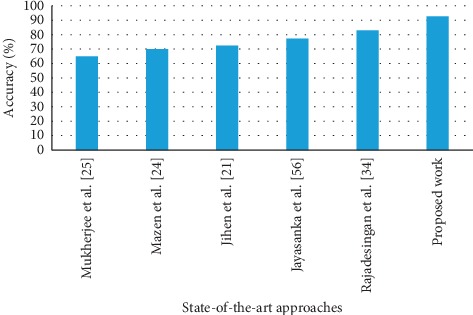
Comparison of the proposed system with existing approaches.

Figure 9 shows the accuracy of the proposed system compared with existing baseline systems. It can be concluded from the graph that the proposed system for sarcasm detection yields a better accuracy than the baseline models.

In Figure 9, the state-of-the-art techniques are compared with the proposed approach for detecting sarcasm. The classification algorithms that are discussed in the above approaches are listed as follows: Naïve Bayes algorithm with fuzzy clustering [23]; maximum entropy and Naïve Bayes approach [49]; Naïve Bayes algorithm [9]; random forest classification [18]; and scuba framework [50]. The comparison of the existing approaches with the proposed work is tabulated in Table 2.

**Figure 10:**
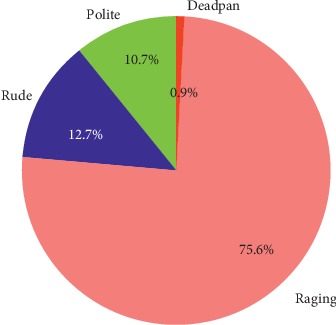
Sarcastic type classification based on proposed multi-rule approach.

Figure 10 shows the different types of sarcasm detected by the proposed multi-rule based approach. In the proposed method, sarcasm was classified into four types, namely, Polite, Rude, Deadpan, and Raging.

Rough set-based analysis of type of sarcasm is performed on the results obtained by the fuzzy system, and the confusion matrix obtained by the rough set is tabulated in Table 3.

As seen from the confusion matrix presented in Table 3, the misclassification of types is very minimal across the four types under study.  Table 4 shows the accuracy obtained through the rough set approach-based validation. As inferred from the confusion matrix, accuracy of classification is high for all the types of sarcasm (Polite—95.98%, Rude—96.2%, Raging—99.79%, and Deadpan—86.61%). The misclassification rate for each type of sarcasm is minimal too with 4.02%, 3.8%, 0.21%, and 13.39% for polite, rude, raging, and deadpan types of sarcasm, respectively.

**Figure 11:**
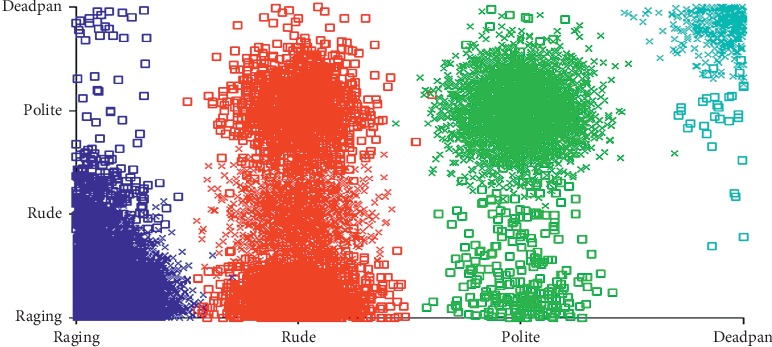
Cluster analysis of type of sarcasm.

Based on the result obtained, a clustering analysis was performed on the sarcastic types and is represented in Figure 11. Performance measure of the proposed system is tabulated in Table 5. Precision, recall, and F-measure were calculated based on equations mentioned in Algorithm 3. It can be inferred that the overall accuracy of 89.1% in rough set analysis affirms the performance of the fuzzy rule base developed for type of sarcasm.

**Figure 12:**
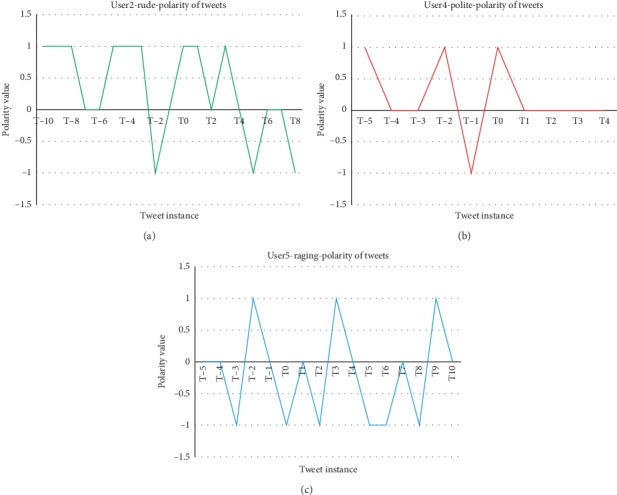
Representation of mood changes of users. (a) User 2 polarity of tweets: Rude. (b) User 4 polarity of tweets: Polite. (c) User 5 polarity of tweets: Raging.

Polarity analysis of preceding and succeeding tweets of a sarcastic tweet for different users is studied, and the results obtained are presented as figures. Figures 12(a)–12(c) depict the change in mood level obtained for users for different types of sarcasm obtained. T0 represents the sarcastic tweet under consideration and the past tweets of T0 are represented as (T−1), (T−2), etc.

The tweets following sarcastic tweet are named as (T1), (T2), (T3), etc. The polarity analysis is carried out for the preceding and succeeding tweets for different users and different types of sarcasm. For polite sarcasm, the polarity of tweets tends to be more positive in nature while for rude and raging sarcasm, the mood of the user tends to be more negative. It matches with the assumption that when exhibiting polite sarcasm, the general mood of the user tends to be positive, and similarly, when exhibiting rude and raging sarcasm, the overall mood of the user tends to be negative as his/her mood tends to be disturbed by anger or rage, which is exhibited by Figure 12.

## 5. Conclusion

Sarcasm is an implicit form of the sentiment. Detecting sarcasm present in the text helps in understanding the actual emotion with which information was conveyed. In order to detect sarcasm, various features are extracted which include positive and negative intensifiers, skip grams, sentiment value, polarity flip, etc. An ensemble-based feature selection method has then been proposed to identify the optimal feature set among all features and to classify whether a tweet is sarcastic or not, and it achieves an accuracy of 92.7%. Apart from classifying a text as sarcastic or nonsarcastic, a novel attempt has been carried out in this work for categorization of sarcasm into various types. Sarcasm has been classified into four types after analyzing the properties of sarcasm in detailed manner, namely, polite, rude, raging, and deadpan. In order to classify sarcasm into various types, a multi-rule based approach has been proposed. The proposed multi-rule based approach consists of two levels: fuzzy rule-based type detection and rough set-based type detection and validation. Fuzzy rules have been constructed for modelling the various types of sarcasm and the same has been validated and verified with rough set theory-based rules. The proposed multi-rule based approach attained the following results for different sarcastic types: Polite, 95.98%; Rude, 96.2%; Raging, 99.79%; and Deadpan, 86.61%. The results obtained have supported the notion that mood levels of the person influence the type of sarcasm they exhibit. When a person is in not so good mood, rude and raging sarcasm tends to be predominant in the outcome exhibited, and when the mood is positive, they tend to exhibit polite sarcasm predominantly. The validation of the results of the type of sarcasm obtained through fuzzy system was carried out with the rough set approach. Finally, after determining the type of sarcasm, user's mood changes were modelled in the proposed approach. To predict how a user's mood influences the sarcasm and vice versa, the tweets before and after a particular exhibition of a sarcastic type are obtained. This can help in modelling the emotion change of a user by collecting the past tweet histories of each user. The obtained mood change model demonstrates how the type of sarcasm exhibited by the users affects their mood levels.

## Figures and Tables

**Algorithm 1:**
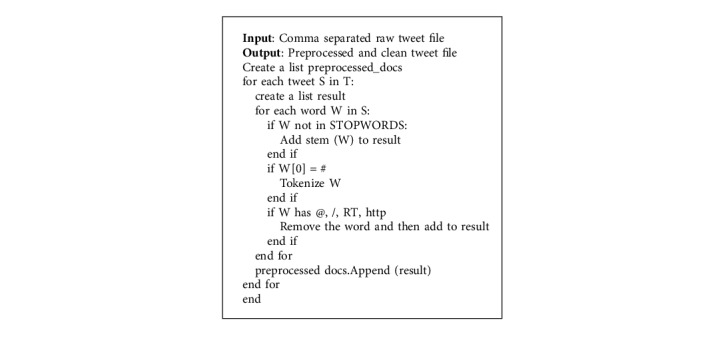
Preprocessing of raw twitter data.

**Algorithm 2:**
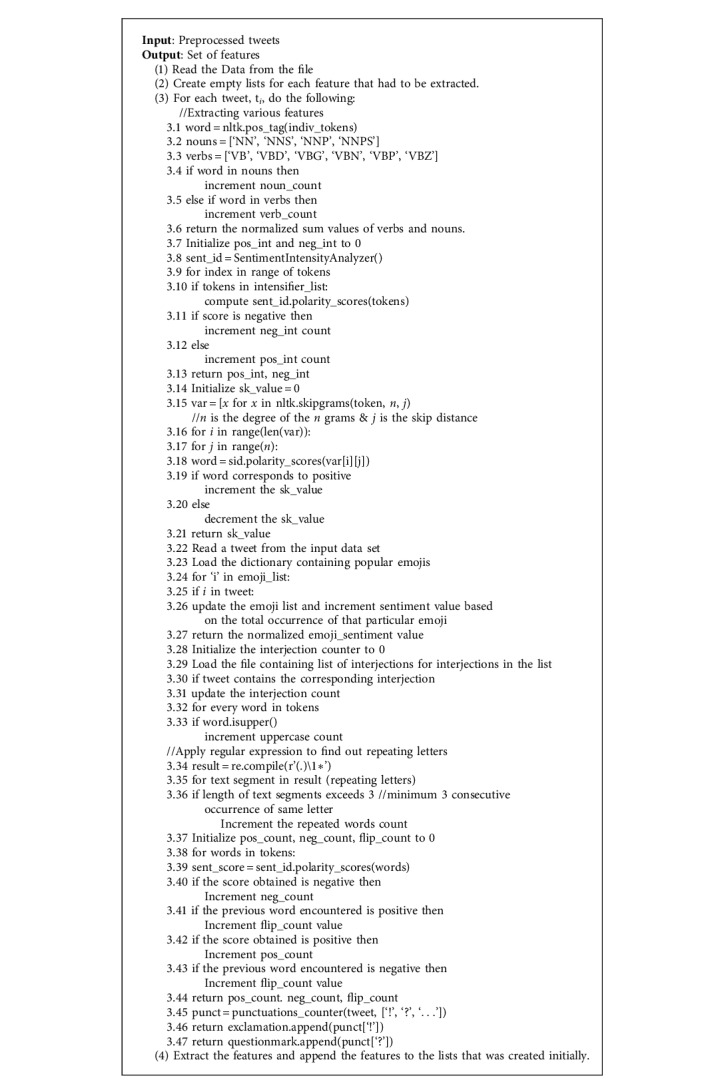
Feature selection.

**Algorithm 3:**
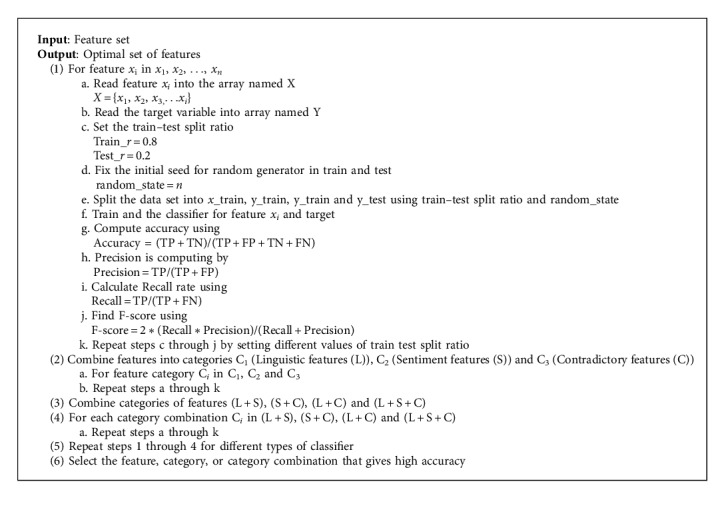
Proposed ensemble feature selection.

**Algorithm 4:**
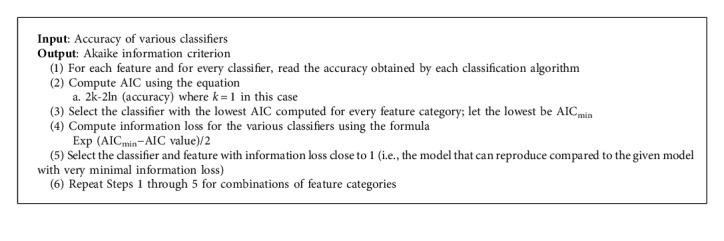
AIC computation.

**Algorithm 5:**
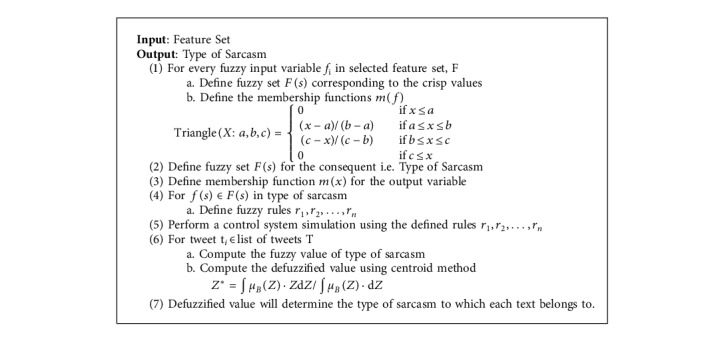
Fuzzy algorithm for sarcastic type detection.

**Algorithm 6:**
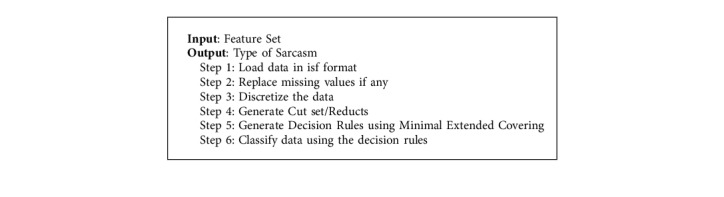
Rough set algorithm.

**Algorithm 7:**
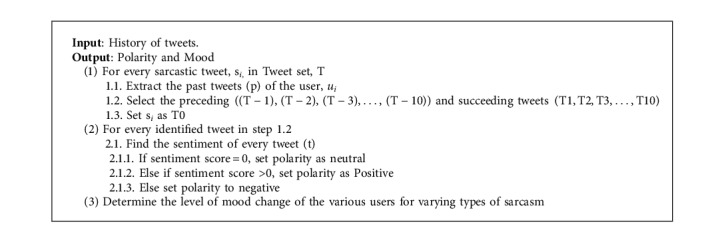
Modelling the user's Mood changes.

**Table 1:** Sample *n*-gram and skip gram feature extraction.

Unigram	{‘it'}	{‘was'}	{‘supposed'}	{‘to'}	{‘be'}	{‘a'}	{‘joke'}	
Bigram	{‘it', ‘was'}	{‘was', ‘supposed'}	{‘supposed', ‘to'}	{‘to', ‘be'}	{‘be', ‘a'}	{‘a', ‘joke'}		
Trigram	{‘it', ‘was', ‘supposed'}	{‘was', ‘supposed', ‘to'}	{‘supposed', ‘to', ‘be'}	{‘to', ‘be', ‘a'}	{‘be', ‘a', ‘joke'}			
1-skip 3-grams	{‘it', ‘was', ‘supposed'}	{‘it', ‘was', ‘to'}	{‘was', ‘supposed', ‘to'}	{‘was', ‘supposed', ‘be'}	{‘supposed', ‘to', ‘be'}	{‘supposed', ‘to', ‘a'}	{‘to', ‘be', ‘a'}	{‘to', ‘be', ‘joke'}

**Table 2:** Comparison of the proposed approach with the state-of-the-art approaches.

State-of-the-art approaches	Accuracy (%)
[23] Mukherjee and Bala	65
[9] El-Masri et al.	70
[18] Karoui et al.	72.36
[49] Jayasanka et al.	77.28
[50] Rajadesingan et al.	83.46
Proposed work	92.7

**Table 3:** Confusion matrix of the proposed approach.

Type	Polite	Rude	Raging	Deadpan
Polite	3290	46	85	7
Rude	66	4018	87	6
Raging	24	23	24725	5
Deadpan	2	6	31	263

**Table 4:** Classification accuracy obtained by the proposed rough set approach.

Average accuracy (%)		
	Correct	Incorrect
Total	98.81	1.19
Polite	95.98	4.02
Rude	96.2	3.8
Raging	99.79	0.21
Deadpan	86.61	13.39

**Table 5:** Performance measure of the proposed system.

Class	TP-rate	FP-rate	Precision	Recall	F-measure
Raging	0.983	0.231	0.93	0.983	0.955
Rude	0.809	0.017	0.729	0.309	0.434
Polite	0.931	0.041	0.730	0.931	0.818
Deadpan	0.913	0.001	0.869	0.913	0.890
Weighted average	0.891	0.181	0.882	0.891	0.874

## Data Availability

The experimental data used to support the findings of this study are available from the corresponding author upon request.
